# Efficacy and Safety of Modified Yunu-Jian in Patients with Periodontitis: A Meta-Analysis

**DOI:** 10.1155/2021/5147439

**Published:** 2021-04-23

**Authors:** Yuanyuan Yue, Meng Gao, Yanru Deng, Jiemin Shao, Yingguang Sun

**Affiliations:** Department of Hospital Pharmacy, Hebei General Hospital, Shijiazhuang, Hebei Province 050000, China

## Abstract

**Background:**

Modified Yunu-Jian (mYJ), a Chinese medicine (CM) formula, is thought to clear heat and nourish yin. Clinically, it is often used to treat oral inflammation. However, its efficacy remains controversial.

**Methods:**

The study aims to evaluate the efficacy and safety of mYJ for treating patients with periodontitis. We searched electronic databases (PubMed, Cochrane Library, Embase, China National Knowledge Infrastructure, Wanfang database, VIP database, and CBM) from inception to December 2020. Only randomized controlled trials investigating modified Yunu-Jian, with or without other medications, against controlled intervention in the treatment of patients diagnosed with periodontitis were included. Both Review Manager 5.3 and Stata 15.0 software were used to analyze the data. The Cochrane Collaborations risk of bias tool was used to assess the quality of the methods.

**Results:**

Thirteen clinical trials, involving 1179 participants, were included in our investigation. The results showed that the combination of mYJ with western medicine improved the total effective rate compared with western medicine alone (RR = 1.17, 95% CI (1.12, 1.23), *P* < 0.00001). The sensitivity analysis and Harbord's test (*P* = 0.255) both showed that the results were statistically robust. Moreover, the periodontal indexes (GI, SBI, PLI, and PD; *P* < 0.00001) of patients with periodontitis were also significantly improved after receiving the combined therapy. No serious adverse reactions were observed in the experimental groups.

**Conclusions:**

Evidence from the meta-analysis suggested that mYJ appeared to be effective and relatively safe for treating periodontitis. Because of the low quality of the methods used in the included RCTs, further studies with larger sample sizes and well-designed models are required to confirm our findings.

## 1. Introduction

Periodontitis is an infectious disease that results in continuous irreversible destruction of alveolar bone, periodontal ligament, and cementum and, subsequently, loss of teeth [1, 2]. Additionally, it is associated with microorganisms and a host-mediated immune response, affecting a considerable proportion of the population of all ages. In recent years, the number of people suffering from periodontitis has increased substantially [3]. Previous studies have suggested that periodontitis correlates with obesity, type II diabetes, metabolic syndrome, and cardiovascular disease [4–7]. Thus, prevention and cure are considered critically important to avoid the occurrence of this systemic disease. Management of periodontitis mainly involves removing pathogenic factors and repairing, regenerating, and maintaining periodontal tissues. Scaling and root planning are recommended to remove deposits (such as plaque and calculus) from the affected teeth [8]. Following mechanical removal, antibiotics may be prescribed to treat the infection; however, they may cause adverse effects [9, 10]. However, according to the concept of etiologic therapy, eradicating the pathogenic factors is the key to curing the disease.

Yunu-Jian (YJ) is a traditional Chinese medicinal formula recorded in the medical classic Chinese book Jingyue Quanshu, also known as Jingyue's Complete Works. It has been extensively used for the treatment of infectious and febrile diseases by reducing stomach-heat and enriching kidney-yin. YJ contains five main ingredients: gypsum, *Radix rehmanniae, R. ophiopogonis, Rhizoma anemarrhenae*, and *Radix achyranthis bidentatae*. According to the TCM syndrome differentiation, Yunu-Jian is often modified (mYJ) by the addition of other Chinese herbs based on a Yunu-Jian decoction. Zee et al. conducted a high quality, randomized, double-blind, placebo-controlled clinical trial on mYJ use as the assistant drug in the nonsurgical treatment of male smokers with chronic periodontitis. In this study, the used methods were described in detail, which included the sequence generation, blinding, and allocation concealment. The results, visualized as graphs, revealed that mYJ might be capable of increasing the radiographic alveolar bone density and lead to an overall improvement in stomach-heat and kidney-yin [11]. Clinically, several studies have shown that mYJ can significantly reduce the symptoms of patients suffering from periodontitis and achieve remarkable efficacy [11–14]; however, there is no statistical support for this conclusion. In this study, we conducted a systematic review and meta-analysis to evaluate the efficacy and safety of mYJ for treating periodontitis and provide a reference for its clinical use.

## 2. Methods

### 2.1. Search Strategy

In this study, seven electronic databases, including PubMed, Cochrane Library, Embase, China National Knowledge Infrastructure (CNKI), Wanfang database, VIP database, and CBM, were searched from the period of their inception to December 15th, 2020. The domain of terms used for each search was presented as follows: periodontitis, periodontal disease, gingivitis, Yunu-Jian, YunvJian, Jade maiden, women decoction, Fair maiden decoction, and randomized clinical trials. A systematic search was performed based on the combination of subject word and random word, with the language of the publications limited to English and Chinese. In addition, to avoid omissions, we conducted a supplemental search of the U.S. National Library of Medicine and the database of Chinese clinical studies. The detailed search strategy for PubMed and CNKI, as an example, is provided in the Supplementary Materials S1

### 2.2. Selection and Exclusion Criteria

Studies were included if they met the following criteria. (1) According to western medicine's diagnostic criteria of the Chinese Stomatology or other diagnostic criteria for periodontitis, all participants were diagnosed with periodontitis [15]. There were no restrictions on the race, age, gender, type of illness, and severity of the disease. (2) The experimental group was treated with mYJ alone or with a combination of mYJ and western medicine or physiotherapy, while the control group was treated with western medicine or physiotherapy. The physiotherapy mainly involved tooth extraction, open-flap debridement, and scaling and root planning. (3) One or more outcomes were measured and had to include the overall clinical efficacy, symptom scores, or adverse events. (4) The study was a randomized controlled trial (RCT).

The following are the exclusion criteria: (1) data in the literature which were not complete or unavailable for statistical analysis and (2) studies that have been repeatedly published by different centers. Only data with complete information were included.

### 2.3. Data Extraction and Quality Assessment

Two investigators independently screened the literature titles and abstracts and then reviewed the full texts according to the predefined inclusion or exclusion criteria. Then, they extracted the following information: first author, year of the publication, sample size, course of treatment, intervention method, follow-up, and outcomes. A third researcher resolved any disagreements.

Two reviewers assessed the risk of bias for the included studies by employing the Cochrane Collaboration's risk of bias tool. The assessed content included random sequence generation; allocation concealment; blinding of subjects, and experimenters; blinding of outcome assessments; incomplete outcome data; selective reporting; other biases. Each study was classified into low risk, unclear, and high risk.

### 2.4. Statistical Analysis

We conducted this meta-analysis by using Review Manager 5.3 and Stata 15.0 software. The relative risk (RR) with a 95% confidence interval (Cl) was calculated for the dichotomous data, while the mean difference (MD) with 95% CI was calculated for the continuous data. Potential publication bias was assessed based on funnel plots and Harbord's test or Egger's test. The trim-and-fill method was used to validate the publication bias further. Moreover, the chi-square and *I*^2^ tests determined statistical heterogeneity among studies. If significant heterogeneity existed (*P* < 0.1 or *I*^2^ > 50%), a random effect model was applied to calculate the pooled results of RR; otherwise, a fixed-effect model was used. A sensitivity analysis was performed by sequentially removing each study individually to investigate potential sources of heterogeneity.

## 3. Results

### 3.1. Characteristics of the Studies

**Figure 1:**
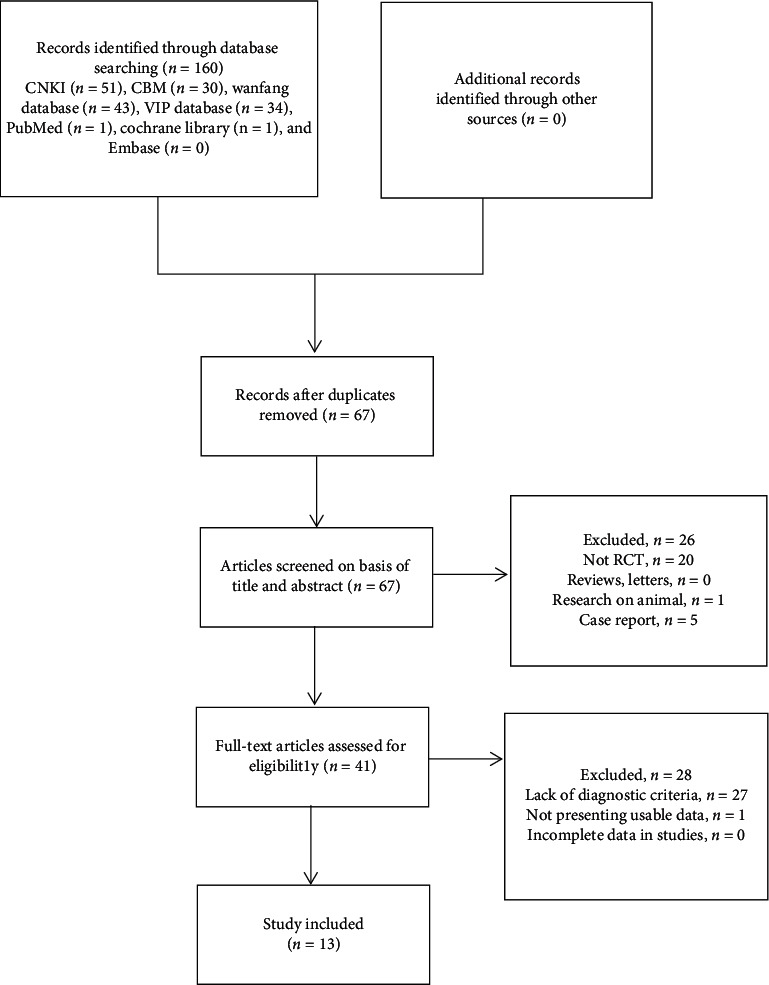
Flow diagram of the study selection process.

One hundred and sixty records were retrieved through the initial search. After further screening, 13 relevant studies involving 1179 patients satisfied the inclusion criteria. A flow diagram of the study selection process is shown in Figure 1. All studies were published in Chinese, and the characteristics of the literature are summarized in Table 1.

### 3.2. Quality Assessment of the Included Studies

**Figure 2:**
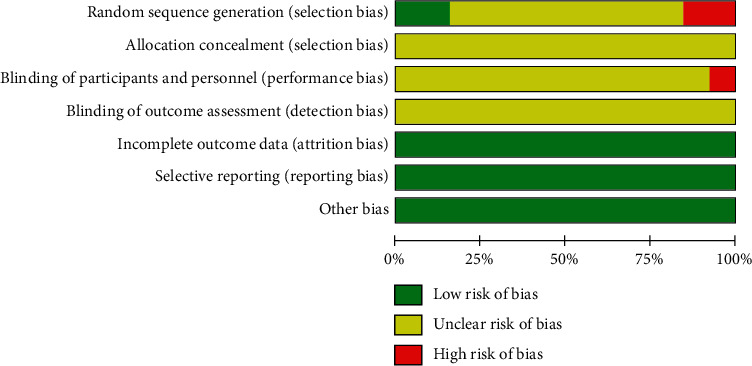
Risk of bias graph.

All the included studies presented no significant differences in the baseline between the experimental and treatment groups. However, only two reported a randomization technique using a random number table. Furthermore, two studies sorted participants into groups following their treatment order, while the other nine did not report the specific information about the randomization technique used. In addition, none of the 13 trials described double-blinding, allocation concealment, dropouts, and follow-ups. Due to the lack of detail and specific information, it cannot be confirmed whether implementations were adequately conducted in the random sequence generation process, blinding, or allocation concealment. As a result, the quality of the methods of the included studies was poor (Figure 2).

### 3.3. Effects of the Interventions

#### 3.3.1. Overall Clinical Efficacy

**Figure 3:**
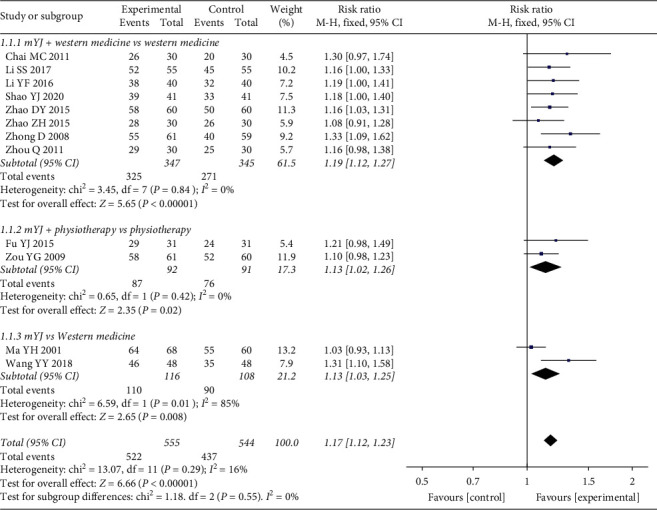
Forest plot of the results of the meta-analysis of the overall efficacy.

Twelve studies containing 1099 patients (555 in the experimental groups versus 544 in the control groups) evaluated overall clinical efficacy [12–14, 16–24]. As the test for heterogeneity was statistically not significant (*P* = 0.29, *I*^2^ = 16%), a fixed-effect model was used for the meta-analysis. The results (Figure 3) indicated that the overall clinical efficacy in the experimental group was significantly better than in the control group (RR = 1.17, 95% CI (1.12, 1.23), *P* < 0.00001).

We performed the subgroup analysis among studies according to the research objectives of the included trials. Eight RCTs tested mYJ plus western medicine against treatment with western medicine alone in patients with periodontitis [12–14, 16–19, 24], and all conducted physiotherapy except for the Zhong study [24]. There was also no heterogeneity among the studies (*P* = 0.84, *I*^2^ = 0%). The outcome showed that the overall clinical efficacy of mYJ combined with western medicine was superior to using western medicine alone for the treatment of periodontitis (RR = 1.19, 95% CI (1.12, 1.27), *P* < 0.00001) (Figure 3, 1.1.1).

Only two RCTs showed that, based on physiotherapy, patients who received mYJ had a higher clinical efficacy compared with those who did not receive the drug (RR = 1.13, 95% CI (1.02, 1.26), *P* = 0.02) [20, 21]. There was a good homogeneity between the two studies (*P* = 0.42, *I*^2^ = 0%) (Figure 3, 1.1.2).

Two trials reported a comparison of mYJ with western medicine in patients with periodontitis [22, 23]. The results of the subgroup analysis showed that mYJ therapy might significantly improve the clinical effect compared with treatment with western medicine (RR = 1.13, 95% CI (1.03, 1.25), *P* = 0.008). However, significant heterogeneity was found between the two studies (*P* = 0.01, *I*^2^ = 85%) (Figure 3, 1.1.3).

#### 3.3.2. Gingival Index (GI)

**Figure 4:**
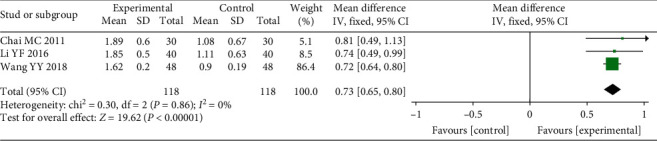
Forest plot of the results of the meta-analysis of the gingival index.

Three of the thirteen included studies reported GI [12, 16, 19, 22]. A fixed-effect model was adopted to estimate the pooled effect sizes (*P* = 0.86, *I*^2^ = 0%) due to good homogeneity. From Figure 4, it can be concluded that the GI of the experimental groups had more obvious improvements compared with the control groups (WMD = 0.73, 95% CI (0.65, 0.80), *P* < 0.00001).

#### 3.3.3. Sulcus Bleeding Index (SBI)

**Figure 5:**
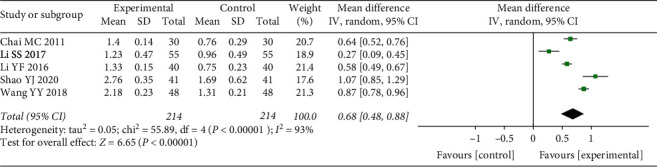
Forest plot of meta-analysis results of sulcus bleeding index.

Five included studies evaluated the SBI [12, 13, 16, 19, 22]. We used a random-effect model for the meta-analysis due to substantial heterogeneity (*P* < 0.00001, *I*^2^ = 93%). As presented in Figure 5, the experimental groups showed a more significant increase in symptom improvement than the control groups (WMD = 0.68, 95% CI (0.48, 0.88), *P* < 0.00001).

#### 3.3.4. Plaque Index (PLI)

**Figure 6:**
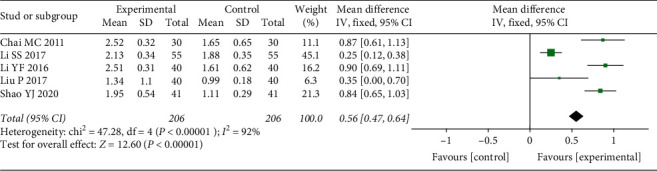
Forest plot of the results of the meta-analysis of the plaque index.

Five qualified pieces of literature reported PLI [12, 13, 16, 19, 25]. A random-effect model was conducted due to the presence of significant heterogeneity (*P* < 0.00001, *I*^2^ = 92%). The results of the meta-analysis showed that a decrease of PLI in the experimental groups was more remarkable than that in the control groups (WMD = 0.56, 95% CI (0.47, 0.64), *P* < 0.00001) (Figure 6).

#### 3.3.5. Probing Pocket Depth (PD)

**Figure 7:**
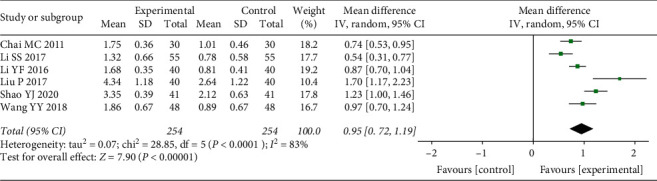
Forest plot of meta-analysis results of pocket depth index.

Of the included trials, six described the situation of PD [12, 13, 16, 19, 22, 25]. There was evidence of poor homogeneity among the studies (*P* = 0.001, *I*^2^ = 83%). Therefore, a random effect model was applied to estimate the pooled effect sizes. The PD of the treatment groups improved significantly when compared with the control groups (WMD = 0.95, 95% CI (0.72, 1.19), *P* < 0.00001) (Figure 7).

### 3.4. Adverse Events

Of all the selected RCTs, only two reported adverse events during the treatment period. Although one study (mYJ versus western medicine) reported that two cases of adverse reactions (one had nausea and the other had vomiting) occurred in the experimental group and eight cases of adverse reactions (three had nausea, one vomited, two had diarrhea, and two had a poor appetite) occurred in the control group [22], these adverse reactions spontaneously disappeared after the treatment session. The Zhao study (mYJ plus western medicine versus western medicine) reported that one patient with chills was found in the experimental group, while three with high fever and five with chills were noted in the control group [17]. However, these adverse reactions did not affect the experimental progress.

### 3.5. Sensitivity Analysis

**Figure 8:**
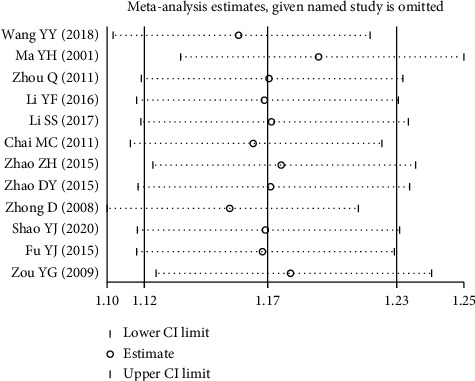
Sensitivity analysis for the primary indicator.

We conducted a sensitivity analysis to evaluate the stability of the primary outcome by removing each study individually. As shown in Figure 8, the sensitivity analysis indicated that no single study significantly affected the primary outcome. As a result, the results of the present study were robust. However, when Ma's study was excluded from the pooled analysis, the studies' homogeneity improved (*P* = 0.68, *I*^2^ = 0%) [23].

### 3.6. Evaluation of the Publication Bias

**Figure 9:**
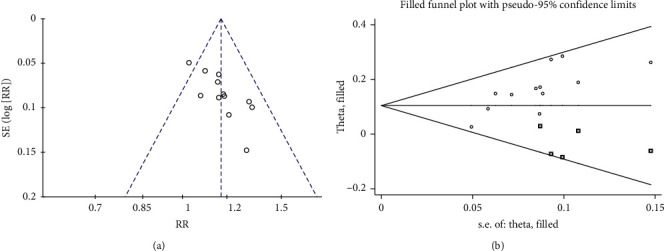
(a) Funnel plot of overall clinical efficacy. (b) Filled funnel plot of overall clinical efficacy.

We used funnel plots and Harbord's test to assess publication bias of the primary outcome. According to Figure 9(a), the funnel plot of overall clinical efficacy was slightly asymmetric, presenting potential publication bias. Harbord's test was used to confirm the publication bias. Moreover, the result showed that the publication bias did not exist in the studies included in the clinical efficacy (*P* = 0.255). The trim-and-fill method was used to determine if the potential bias affected the meta-analysis (Figure 9(b)). The pooled RR with 95% CI did not change significantly before and after the use of the trim-and-fill method (fixed effect model: RR 1.141, 95% CI (1.092, 1.191), *P* < 0.0001 vs. RR 1.106, 95% CI (1.063, 1.150), *P* < 0.0001; random-effect model: RR 1.144, 95% CI (1.093, 1.197), *P* < 0.0001 vs. RR 1.110, 95% CI (1.057, 1.165), *P* < 0.0001) (Figures 9 and Supplementary Materials S4). Accordingly, the slight publication bias did not affect the results of our pooled analysis, proving that our data were robust.

## 4. Discussion

Recently, Chinese medicines have been increasingly used in many countries, especially to treat cancer and chronic conditions, such as functional dyspepsia, diabetes, and periodontitis [26–28]. Some studies demonstrated that *Guchi* pills, *Guchi* extract, *Yazhou baidu,* and mYJ are potential treatments for periodontitis [29]. However, no systematic study has demonstrated how mYJ performs against other medicines for the treatment of periodontitis. This study assessed the efficacy and safety of mYJ in patients with periodontitis. Both Review Manager 5.3 and Stata 15.0 software were employed to analyze clinical data from included RCTs. The meta-analysis, including 13 studies with 1179 participants, suggested that mYJ exerted a positive effect on periodontitis and significantly improved the clinical periodontal indexes of patients (GI, SBI, PLI, and PD). Additionally, the results of the subgroup analysis revealed that the combination therapy using mYJ appeared to be more effective than monotherapy. Moreover, mYJ might have fewer adverse events than western medicine.

In the theory of traditional Chinese medicine, periodontal disease is related to disorders of the kidney, spleen, and stomach, which are caused by various factors, such as poor oral hygiene, stomach-heat, kidney-yin deficiency, weakness in qi, and blood [30, 31]. Moreover, the concept of traditional Chinese medicine dictates that teeth essentially belong to one part of the bone, which is regulated by the kidney. Consequently, the teeth are also dominated by the kidney. Meanwhile, the spleen and stomach meridians pass through the teeth and gum. In general, periodontal disease could be associated with unwanted changes to the homeostasis in the spleen and stomach or kidney [32]. Although mechanical removal is recommended for removing deposits from the affected teeth [9], etiological treatment remains the key to curing the disease.

Modified Yunu-Jian (mYJ), a Chinese medicinal formula, contains five mineral and herbal medicines and has been used to decrease stomach-heat and nourish kidney-yin. Gypsum exerts heat-clearing and fire-purging effects, which can also decrease vascular permeability [33]; *Rhizoma anemarrhenae* has been reported to exhibit a protective effect by inhibiting the action of cerebral ischemia [34]. Some studies have proved that *Radix rehmanniae* inhibits blood platelet aggregation and has antioxidant and anti-inflammatory effects [35]; *Radix ophiopogonis* can enhance endothelial cell protective and antiadhesive activities [36]. *Rhizoma drynariae* and *Rehmannia glutinosa* have also been reported to improve the mending of fractures [37, 38]. The major ingredients of mYJ could reduce in vivo inflammation, enhance immune function, and modulate bone homeostasis [29]. Moreover, its activity can be modified or refined by adding Chinese herbs and/or minor changes to one of its ingredients. mYJ is commonly used in the clinical treatment of diabetes and inflammation of the gums [39, 40].

Although our analysis was conducted using a standardized process, it has some potential limitations. First, all the included studies were translated from written Chinese and obtained from China, introducing an unavoidable regional bias. Second, only two studies described the randomization method, and none of the trials provided information about allocation concealment and blinding, which weakened the credibility of the research. Third, the number of included studies and sample size were both small, and thus, our results might have an inherent bias. Fourth, there were no uniform standards for dosage and course of treatment in the included studies, and none of the studies considered these possible influential factors. Those limitations might impact our conclusion of this meta-analysis.

In conclusion, the evidence from this meta-analysis suggests that mYJ appears to be effective and relatively safe in treating patients with periodontitis. However, because of the poor quality of the methods and small sample size of the included RCTs, further studies with larger sample sizes and well-designed models are required to confirm our findings.

## Figures and Tables

**Table 1:** The characteristics of the included studies.

Study	Sample size	Intervention		Duration/days	Outcome measures
	(E/C)	Experimental group	Control group		
Chai and Qiang [12]	30/30	Physiotherapy + western medicines + mYJ	Physiotherapy + metronidazole + levofloxacin	60	①②③④⑤
Li et al. [13]	55/55	Physiotherapy + western medicines + mYJ	Physiotherapy + minocycline	28	①③④⑤
Zhou and Liu [14]	30/30	Physiotherapy + western medicines + mYJ	Physiotherapy + metronidazole	10	①
Li and Li [16]	40/40	Physiotherapy + western medicines + mYJ	Physiotherapy + metronidazole + roxithromycin	7	①②③④⑤
Zhao [17]	60/60	Physiotherapy + western medicines + mYJ	Physiotherapy + metronidazole + acetylspiramycin	30	①
Zhao [18]	30/30	Physiotherapy + western medicines + mYJ	Physiotherapy + metronidazole	28	①③④⑤
Shao and Sheng [19]	41/41	Physiotherapy + western medicines + mYJ	Physiotherapy + minocycline	28	①③④⑤
Fu [20]	31/31	Physiotherapy + mYJ	Physiotherapy	10	①
Zou et al. [21]	61/60	Physiotherapy + mYJ	Physiotherapy	10	①
Wang [22]	48/48	Physiotherapy + mYJ	Physiotherapy + metronidazole + ibuprofen	7	①②③④
Ma and Qin [23]	68/60	mYJ	Metronidazole + tetracycline	7	①
Zhong and Li [24]	61/59	Western medicine + mYJ	Metronidazole + acetylspiramycin	10	①
Liu and Li [25]	40/40	Western medicine + mYJ	Omidazole	14	④⑤

## Data Availability

The data used to support the findings of this study are included within the article and the supplementary information files.

## Supplementary Materials

Supplementary Materials S1: retrieval strategy for PubMed and CNKI. S2: PRISMA checklist. S3: Harbord's test for clinical efficiency. S4: the trim-and-fill method for funnel plot of total effective rate.
