# Supplementary material for: Efficacy and Safety of Modified Yunu-Jian in Patients with Periodontitis: A Meta-Analysis
Source: Evid Based Complement Alternat Med. 2021 Apr 23;2021:5147439. doi: 10.1155/2021/5147439 (PMC8093059; doi:10.1155/2021/5147439)
Supplement: Supplementary Materials — S1: retrieval strategy for PubMed and CNKI. S2: PRISMA checklist. S3: Harbord's test for clinical efficiency. S4: the trim-and-fill method for funnel plot of total effective rate. [file 5147439.f1.zip › 5147439.f1/S1-Retrieval strategy for PubMed and CNKI.doc]

1. PubMed retrieval strategy

| search | query |
| --- | --- |
| #1 | Yunvjian[Title/Abstract] |
| #2 | Yunujian[Title/Abstract] |
| #3 | “Jade maiden decoction”[Title/Abstract] |
| #4 | “women decoction”[Title/Abstract] |
| #5 | “Fair maiden decoction”[Title/Abstract] |
| #6 | #1OR #2 OR #3 OR #4 OR #5 |
| #7 | Periodontitis[MeSH] |
| #8 | Periodontal inflammation[Title/Abstract] |
| #9 | Periodontitis[Title/Abstract] |
| #10 | #7 OR #8 OR #9 |
| #11 | #6 AND #10 |

2. CNKI retrieval strategy

SU % 'yunujian' AND( SU % 'periodontitis' OR SU % 'periodontal disease' OR SU % 'gingivitis')
