# Supplementary material for: Efficacy and Safety of Modified Yunu-Jian in Patients with Periodontitis: A Meta-Analysis
Source: Evid Based Complement Alternat Med. 2021 Apr 23;2021:5147439. doi: 10.1155/2021/5147439 (PMC8093059; doi:10.1155/2021/5147439)
Supplement: Supplementary Materials — S1: retrieval strategy for PubMed and CNKI. S2: PRISMA checklist. S3: Harbord's test for clinical efficiency. S4: the trim-and-fill method for funnel plot of total effective rate. [file 5147439.f1.zip › 5147439.f1/S3-Harbord's test for clinical efficiency.doc]

The Harbord’ s test for clinical efficiency

Harbord's modified test for small-study effects:

Regress Z/sqrt(V) on sqrt(V), where Z is the efficient score and V is the score variance

.

Number of studies = 12	Root MSE	=	.5276

------------------------------------------------------------------------------

Z/sqrt(V) |	Coef. Std. Err. t	P>|t|	[95% Conf. Interval]

-------------+----------------------------------------------------------------

sqrt(V)|	1.903788	.5585675	3.41	0.007	.6592223	3.148354

|

bias | -1.071626	.8874014	-1.21	0.255	-3.048879	.9056279

------------------------------------------------------------------------------

Test of H0: no small-study effects	P = 0.255
