# Supplementary material for: Efficacy and Safety of Modified Yunu-Jian in Patients with Periodontitis: A Meta-Analysis
Source: Evid Based Complement Alternat Med. 2021 Apr 23;2021:5147439. doi: 10.1155/2021/5147439 (PMC8093059; doi:10.1155/2021/5147439)
Supplement: Supplementary Materials — S1: retrieval strategy for PubMed and CNKI. S2: PRISMA checklist. S3: Harbord's test for clinical efficiency. S4: the trim-and-fill method for funnel plot of total effective rate. [file 5147439.f1.zip › 5147439.f1/S4-The trim and fill method for funnel plot of total effective rate (1).doc]

The trim-and-fill method for funnel plot of overall clinical efficacy.

Meta-analysis

| Pooled 95% CI Asymptotic No. of

Method |	Est	Lower	Upper z_value p_value	studies

-------+----------------------------------------------------

Fixed |	0.132	0.088	0.175	5.940	0.000	12

Random |	0.134	0.089	0.180	5.799	0.000

Test for heterogeneity: Q= 11.751 on 11 degrees of freedom (p= 0.383)

Moment-based estimate of between studies variance = 0.000

Trimming estimator: Linear

Meta-analysis type: Random-effects model

iteration | estimate	Tn	# to trim	diff

----------+--------------------------------------

1	|	0.134	55	3	78

2	|	0.110	65	5	20

3	|	0.101	67	5	4

4	|	0.101	67	5	0

Filled

Meta-analysis (exponential form)

| Pooled 95% CI Asymptotic No. of

Method |	Est	Lower	Upper z_value p_value	studies

-------+----------------------------------------------------

Fixed |	1.106	1.063	1.150	5.048	0.000	17

Random |	1.110	1.057	1.165	4.185	0.000

Test for heterogeneity: Q= 23.174 on 16 degrees of freedom (p= 0.109)

Moment-based estimate of between studies variance = 0.003

Meta-analysis (exponential form)

| Pooled 95% CI Asymptotic No. of

Method |	Est	Lower	Upper z_value p_value	studies

-------+----------------------------------------------------

Fixed |	1.141	1.092	1.191	5.940	0.000	12

Random |	1.144	1.093	1.197	5.799	0.000

Test for heterogeneity: Q= 11.751 on 11 degrees of freedom (p= 0.383)

Moment-based estimate of between studies variance = 0.000
